# Long-term enclosure at heavy grazing grassland affects soil nitrification via ammonia-oxidizing bacteria in Inner Mongolia

**DOI:** 10.1038/s41598-022-25367-z

**Published:** 2022-12-12

**Authors:** Qing Chen, Yuntao Shang, Rui Zhu, Qiongli Bao, Shan Lin

**Affiliations:** 1 Tianjin Key Laboratory of Water Resources and Environment, Tianjin Normal University, Tianjin, China; 2 Agro-Environmental Protection Institute, Ministry of Agriculture and Rural Affairs, Tianjin, 300191 China; 3 College of Resource and Environmental Sciences, China Agricultural University, Beijing, China

**Keywords:** Biogeochemistry, Ecology, Grassland ecology, Microbial ecology

## Abstract

Enclosure and grazing can significantly change the turnover of nitrogen in grassland soil. Changes of soil nitrogen mineralization and ammonium-oxidizing microorganisms caused by enclosure in different grazing intensities (about 30 years of grazing history) grassland, however, has rarely been reported. We selected the grassland sites with high and medium grazing intensity (HG and MG, 4 and 2 sheep ha^−1^, respectively) and had them enclosed (45 × 55 m) in 2005 while outside the enclosure was continuously grazed year-round. A two factorial study was designed: grazing intensity (MG and HG sites) and enclosure (fence and non-fence). Nitrogen mineralization was detected through a laboratory incubation experiment. The abundance and community structure of soil ammonia-oxidizing archaea (AOA) and ammonia-oxidizing bacteria (AOB) were analyzed using quantitative PCR (q-PCR), terminal-restriction fragment length polymorphism (T-RFLP), cloning, and sequencing. Results showed that compared with MG site, at HG site the AOB abundance and community structure of AOB changed significantly while the AOA abundance and community structure did not change obviously. Enclosure significantly decreased the cumulative mineralized N, N mineralization rate, the abundance of AOB and the AOB community structure at the HG site, while at MG site, enclosure did not change these parameters. Potential nitrification rate (PNR) was positively correlated with the abundance of AOA and AOB at the MG and HG sites, respectively. The abundance of AOA was significantly correlated with soil pH; however, AOB abundance was significantly correlated with soil available N, total N, C/N ratio, pH, etc. The phylogenetic analysis showed that Nitrososphaeraceae and Nitrosomonadaceae were the dominant AOA and AOB, respectively. Totally, the responses of AOB and AOA mainly were associated to changes in soil physicochemical properties caused by different intensity grazing; AOB and AOA may be the dominant functional players in ammonia oxidation processes at HG and MG site, respectively.

## Introduction

With important ecological functions and economic value, inner Mongolia grassland is one of the largest and well-preserved natural grasslands in the world. At present, overgrazing has altered the nutrient cycle of the grassland ecosystem, resulting in nutrient loss, grassland degradation, and lead to variations in both microbial diversity and the potential functioning of micro‑organisms^1–3^. As an important means of restoring degraded grassland, enclosures have been widely used to prevent over-exploitation^4,5^.

Nitrogen is widely considered as the principal growth-limiting nutrient for plant and microorganisms in soil^6^. The change of land use patterns significantly affects the nitrogen cycling and the availability of nitrogen in grassland^7^, further affecting the productivity level and function of grassland ecosystems. Soil N mineralization, as an important ecological process, determines soil nitrogen supply capacity^8,9^. Previous studies have detected the influences of grazing on soil mineralizable N (Nmin) and have found that grazing can stimulate the accumulation of Nmin in typical grasslands of Inner Mongolia^10^, and moderate grazing results in the largest accumulation of available N^11^. Synthesis data from multiple studies on grazed Northern Great Plains ecosystems also showed that grazing enhanced soil Nmin^12^; however, the influence of enclosures on Nmin is uncertain. Enclosures significantly increased soil Nmin in temperate grassland of Hulunbeir, Inner Mongolia and Kobresia alpine grassland, Tibet^13,14^; however; it was found that enclosures nearly had no significant effect on soil Nmin in the semi-arid steppe in central Argentina^15^. These differences of the findings could be attributed to dissimilar grassland types, soil characteristics, or microbial effects.

Nitrification plays an important role in the nitrogen cycle, which determines the effectiveness of nitrogen on plants^16–18^. Ammonia oxidation is the rate limiting step of nitrification process, which was considered to be performed by ammonia-oxidizing archaea (AOA) and ammonia-oxidizing bacteria (AOB)^16^. It was found that the change of grassland use pattern had different effects on the abundance and community structure of ammonia oxidizing microorganisms and then affected the nitrogen cycle^19,20^. In typical grasslands of Inner Mongolia, AOA varied with the change of grassland management (grazing and mowing), while abundance and community structure of AOB remained unchanged under different grassland management^19^. However, in the desert steppe of Inner Mongolia, grazing did not affect the abundance of AOA or community structure of AOB but significantly reduced the abundance of AOB as well as affected the community structure of AOA^14^. Many studies on the effects of grazing and enclosures on soil Nmin and microorganisms have been carried out and the findings vary due to the differences in environmental factors. However, there are few studies on the effects of enclosure in different grazing intensities sites (long grazing history for about 30 years) in soil Nmin and ammonia-oxidizing microorganisms in the typical grasslands of Inner Mongolia.

This study mainly focused on the influences of the enclosure in different grazing intensities (moderate grazing (MG) and heavy grazing (HG)) sites on soil Nmin and ammonia-oxidizing microorganisms. The results of study are of great significance in understanding the nitrogen turnover mechanism in grassland with enclosure management in different grazing intensity sites.

## Materials and methods

### Description of site

The study was conducted at the Inner Mongolia Grassland Ecosystem Research Station (IMGERS) in the Xilin River Basin (43°26′–44°29′ N, 115°32′–117°12′ E), Inner Mongolia, China. The average annual rainfall is 343 mm, and 60–80% of precipitation occurs from May to late August^21^. The mean annual temperature is 0.7 °C, with the highest monthly average of 19.0 °C in July, and the lowest monthly average of −21 °C in January. We selected two representative sites, which differed in stocking rate over the past 30 year (from 1970s): (a) moderately grazed (MG, 2 sheep ha^−1^) and (b) heavily grazed (HG, 4 sheep ha^−1^). In this area of Inner Mongolia, these grazing patterns are typical. Soil physicochemical properties of the two sites were shown in Table 1, and soil is calcic chernozems.

**Table 1:** Soil physicochemical properties of experimental site with moderately grazed history (MG) and site with heavily grazed management (HG).

	MG	HG	MG		HG	
			NF	F	NF	F
pH	**7.29 ± 0.06 B**	**8.06 ± 0.11 A**	7.41 ± 0.02 a	7.17 ± 0.09 a	8.15 ± 0.14 *a*	7.97 ± 0.08 *a*
SOC (g kg^−1^)	**11.13 ± 0.55 A**	**9.56 ± 0.44 B**	11.00 ± 0.28 a	11.27 ± 0.82 a	9.49 ± 0.36 *a*	9.63 ± 0.52 *a*
TN (g kg^−1^)	**1.10 ± 0.03 A**	**0.79 ± 0.07B**	1.03 ± 0.03 b	1.16 ± 0.03 a	0.76 ± 0.06 *a*	0.82 ± 0.09 *a*
TP (g kg^−1^)	0.19 ± 0.01 A	0.19 ± 0.02 A	0.19 ± 0.01 a	0.19 ± 0.01 a	0.20 ± 0.01 *a*	0.18 ± 0.02 *a*
C/N	**10.15 ± 0.36 B**	**12.25 ± 0.71 A**	10.70 ± 0.22 a	9.70 ± 0.50 a	12.60 ± 0.97 *a*	11.90 ± 0.92 *a*
N/P	**5.76 ± 0.16 A**	**4.25 ± 0.13B**	**5.47 ± 0.13 b**	**6.05 ± 0.19 a**	**3.80 ± 0.15 *b***	**4.65 ± 0.11 *a***
Water content (%)	**3.79 ± 0.10 A**	**2.01 ± 0.12B**	3.64 ± 0.06 a	3.94 ± 0.18 a	1.95 ± 0.06 *a*	2.07 ± 0.24 *a*
C_NO3-N_ (mg kg^−1^)	**0.83 ± 0.07 B**	**1.51 ± 0.28 A**	0.90 ± 0.05 a	0.75 ± 0.08 a	**2.25 ± 0.85 *a***	**0.76 ± 0.10 *b***
C_NH4+-N_ (mg kg^−1^)	2.28 ± 0.3 A	1.78 ± 0.34 A	2.18 ± 0.19 a	2.38 ± 0.4 a	2.20 ± 0.52 *a*	1.36 ± 0.16 *a*
Available N (mg kg^−1^)	3.10 ± 0.29 A	3.29 ± 0.8A	3.07 ± 0.19 a	3.13 ± 0.39 a	**4.46 ± 1.35 *a***	**2.12 ± 0.25 *b***

To compare the effects of heavy grazing and moderate grazing on soil physical and chemical properties with or without enclosure condition, the average values of NF and F under MG/HG condition were calculated and showed in the first two columns of “MG”/“HG”. NF, non-fenced; F, fenced treatment. Different normal and italic lowercase letters indicate differences between non-fenced (free grazing) and fenced treatment at the MG and HG sites, respectively (*p* < 0.05, n = 4). Different capital letters indicate differences between the MG and HG sites across non-fenced and fenced treatment (*p* < 0.05, n = 8).

*TN* total N concentration, *TP* total phosphorus concentration, *SOC* soil organic carbon concentration, *C*_*NO3-N*_ NO_3_^−^-N concentration, *C*_*NH4+-N*_ NH_4_^+^-N concentration.

### Experimental design

At the beginning of May 2005, a 45 × 55 m area was enclosed at both the MG and HG sites. Outside the enclosure, the grassland was grazed continuously all year (4 and 2 sheep ha^−1^, respectively). Therefore, a two factorial experiment was designed in this study: grazing patterns (MG and HG sites) and enclosure (fence and non-fence), with four replicates. At the end of August, vegetation in all enclosure plots was cut to 5 cm in height and removed from the site.

### Soil sampling for N mineralisation incubation experiment

To minimize the effect on experimental plots, we only extracted undisturbed soil samples in the four enclosure plots and the four random points outside the enclosure at both the MG and HG sites. PVC cylinders (17 cm in length, 5 cm in diameter), with one side sharpened, were hammered 15 cm into the soil to extract undisturbed soil cores for the incubation experiment. Twelve soil cores (6 incubation periods × 2 water levels) were taken in each subplot. A total of 192 soil cores (2 fence levels × 2 sites × 4 replications × 6 incubation periods × 2 water levels) were taken for incubation. The PVC cores were covered with the Parafilm to reduce water loss but allow gas exchange during transport. These soil cores were incubated at a constant temperature of 25 ℃ and soil moisture of 5 and 10 g H_2_O/100 g in the laboratory with four replications^22^. Constant soil moisture was maintained by adding water based on the weighing method every 3 days. The six soil cores from the same subplot were randomly assigned to one of six different incubation periods of 3, 7, 14, 28, 56, 112 days. After incubation, the soil cores were sieved (2 mm mesh) and extracted with 0.01 M CaCl_2_, and then the extracts were used to analyze ammonium (NH_4_^+^-N) and nitrate (NO_3_^–^ N) through Continuous Flow Analysis (Auto Analyzer 3, Nordstadt, Germany).

### Soil sampling for microorganism and potential nitrification rate (PNR) measurement

Soil samples for microorganism measurement were taken from the same plots as the soil N mineralization incubation experiment (total 16 sample: 2 fence levels × 2 sites × 4 replications) in 2014. Five drills samples with diameters of 2 cm were randomly sampled from 0 to 15 cm of soil at each sampling subplots, and five drills were mixed into one sample and passed through 2 mm sieves. Stones, plant residue, and roots in soil samples were removed. The soil sample was selected using the ‘quartering method (the process of mixing the sample evenly and then dividing the sample by a ratio of 2/4 is called quartering)’, and fresh soil samples were stored at 4 °C during transportation. Each fresh soil sample was separated into three sub-samples: one sub-sample was preserved at 4 °C for determine nitrification potential within 48 h; one sub-sample was stored at −80 °C for microbial analysis within a week; the third sub-sample was air-dried to measure soil physicochemical properties within a week.

#### Soil physicochemical properties analysis

Air-dried soils were sieved (0.25 mm mesh) before physicochemical properties were measured. The soil pH (soil:water = 1:5) was measured using a conductivity meter (Thermo Orion, United States). The soil organic carbon (SOC) and total nitrogen (TN) were measured using an elemental analyzer (Elementar, Germany). Total phosphorus (TP) was determined colourimetrically after wet digestion with H_2_SO_4_ and HClO_4_. The soil physicochemical properties are showed in Table 1.

#### Soil potential nitrification rate (PNR)

The chlorate inhibition method^23^ was used for measuring PNR. For the assay, 5 g of fresh soil was added to 50 mL centrifuge tubes, which containing 20 mL of phosphate buffer solution (PBS) (g·L^−1^: NaCl, 8.0; KCl, 0.2; Na_2_HPO_4_, 0.2; NaH_2_PO_4_, 0.2; pH 7.4) with a final concentration of 1 mM (NH_4_)_2_SO_4_. KClO_3_ 10 mM was added for inhibiting nitrite oxidation. The soil slurry was incubated at 25 °C for 24 h, and then nitrite was extracted with 5 mL of 2 M KCl, and shaken extracts were measured using a continuous flow analyzer.

#### DNA extraction and purification

DNA was extracted using the WelPrep DNA kit (Welgene Biotech Co., Ltd) according to the manufacturer’s instructions^24^. The extracted DNA was quantified using a UV–Vis Spectrophotometer (ND-1000, NanoDrop, USA).

#### Quantitative PCR (q-PCR) for *amoA* gene

The AOA and AOB *amoA* gene copy numbers were quantified by q-PCR using a 7300 Real-Time PCR System (Applied Biosystems, Foster City, CA, USA). Primers and thermal cycling conditions used for q-PCR are listed in Table S1. Product specificity was checked through melting curve analysis and agarose gel electrophoresis. The standard curves were established using *amoA* gene fragments cloned into a plasmid pGEM-T Easy Vector (3015 bp, Promega Madison, USA). Positive clones were extracted using a Plasmid Mini Kit (Qiagen Nordic). The AOA and AOB *amoA* gene copy numbers were calculated from the concentrations of the corresponding extracted plasmid DNA with concentrations ranging from 1.732 × 10^1^ copies µL^−1^ to 1.732 × 10^8^ copies µL^−1^ and 2.384 × 10^1^ copies µL^−1^ to 2.384 × 10^6^ copies µL^−1^, respectively. The efficiency of q-PCR was 102.3% (r^2^ = 0.992) for AOA and 105.2% (r^2^ = 0.995) for AOB.

#### PCR amplification and T-RFLP (terminal restriction fragment length polymorphism) analysis of *amoA* gene

The T-RFLP method was used to evaluate the effects of grazing and enclosure on ammonia-oxidizing microbial community structure, and the PCR amplification was performed using the same primer pairs as the q-PCR assays (Table S1), with the forward primer labeled with 6-carboxyfluorescein. The 25 μl PCR reactions included 2.5 μl 10 × PCR buffer (Mg^2+^ plus), 2 μl 2.5 mM dNTPs, 0.25 μl Ex Taq HS polymerase (5 U μl^−1^, Takara Biotechnology, Dalian, China), 0.5 μl of each primer, and 2 μl diluted DNA template. PCR products were gel-purified using PCR Clean-Up System (Promega, USA). The PCR products were digested with the restriction enzyme FastDigest^®^ Mbo I (Takara Biotechnology, China) at 37 °C for 5 min and subsequently at 65 °C for 15 min. The digested products were purified by ethanol precipitation and mixed with deionised formamide, and then determined with an ABI PRISM 3130XL Genetic Analyzer (Applied Biosystems).

#### Phylogenetic analysis

The *amoA* genes of AOA and AOB were constructed from both the HG and MG sites in the fenced treatment soil by using the same primer pairs as q-PCR. PCR was performed by mixing DNA from three duplicate soil samples in each treatment. The PCR products were purified and ligated into the pGEM-T Easy Vector (Promega, Madison, WI). Plasmids were transformed into *Escherichia coli* JM109 (Takara Biotechnology Company, China). 166 and 100 positive clones from each treatment were selected for *amoA* genes of archaeal and bacterial, respectively, and were sequenced by an ABI 3730 sequencer (Applied Biosystem, USA). The operational taxonomic unit (OTU) defined at 96% similarity was estimated using Clustal X (1.83). A representative sequence of each OTU and the related sequences obtained from the NCBI database were used for constructing the phylogenetic tree with MEGA 5.0 using the neighbour-joining method.

#### Calculations and statistical analysis

The cumulative mineralized soil N (Nmin) and Soil net N mineralization rates (Rmin) were calculated according to the following formulas:

$$\mathrm{Nmin}_{{\rm i}+1} = \left(\mathrm{NH}_{4}^ {+}- \mathrm{N}_{{\rm i}+1} +\mathrm{ NO}_{3}^{-} -\mathrm{N}_{{\rm i}+1}\right)$$

$$\mathrm{Rmin }= [(\mathrm{NH}_{4}^{+}-\mathrm{N}_{{\rm i}+1} -\mathrm{ NH}_{4}^{+}-\mathrm{N}_{\rm i}) + (\mathrm{NO}_{3}^{-}-\mathrm{N}_{{\rm i}+1} -\mathrm{ NO}_{3}^{-} -\mathrm{N}_{\rm i})] / (\mathrm{t}_{{\rm i}+1} -\mathrm{ t}_{\rm i})$$

where t_i_ and t_i+1_ are the beginning and end dates of each incubation period, respectively. NH_4_^+^-N_i_, NO_3_^−^-N_i_ and NH_4_^+^-N_i+1_, NO_3_^−^-N_i+1_ were the concentrations of soil NH_4_^+^-N and NO_3_^–^N in the initial and incubated samples, respectively^25^.

All statistical analyses were performed using SPSS Version 17.0 for Windows (SPSS Inc., Chicago, Illinois). The significance of treatment effects and their interactions on the observed parameters were detected through a three-way analysis of variance (ANOVA). Quantitative differences between treatments were examined using a least-significant difference (LSD) test. Figures were generated using the Origin 8.0 package (Origin Lab Corporation, USA).

### Ethical approval

All authors declared that they had no known competing financial interests or personal relationships that seemed to affect the work reported in this article. All authors followed the ethical responsibilities of this journal.

## Results

### Soil properties

Heavy grazing significantly decreased SOC, TN, N/P, and water content but significantly increased soil pH and C/N compared with those at the MG site (Table 1). Long-term enclosure did not significantly change soil pH, SOC, TP, C/N, and water content but significantly increased soil N/P at both sites (Table 1). Long-term enclosure significantly increased soil TN at the MG site, while it significantly decreased the soil NO_3_^-^ -N concentration at the HG site (Table 1).

### Cumulative soil mineralized N (Nmin) and N mineralization rate (Rate_Nmin)

**Figure 1:**
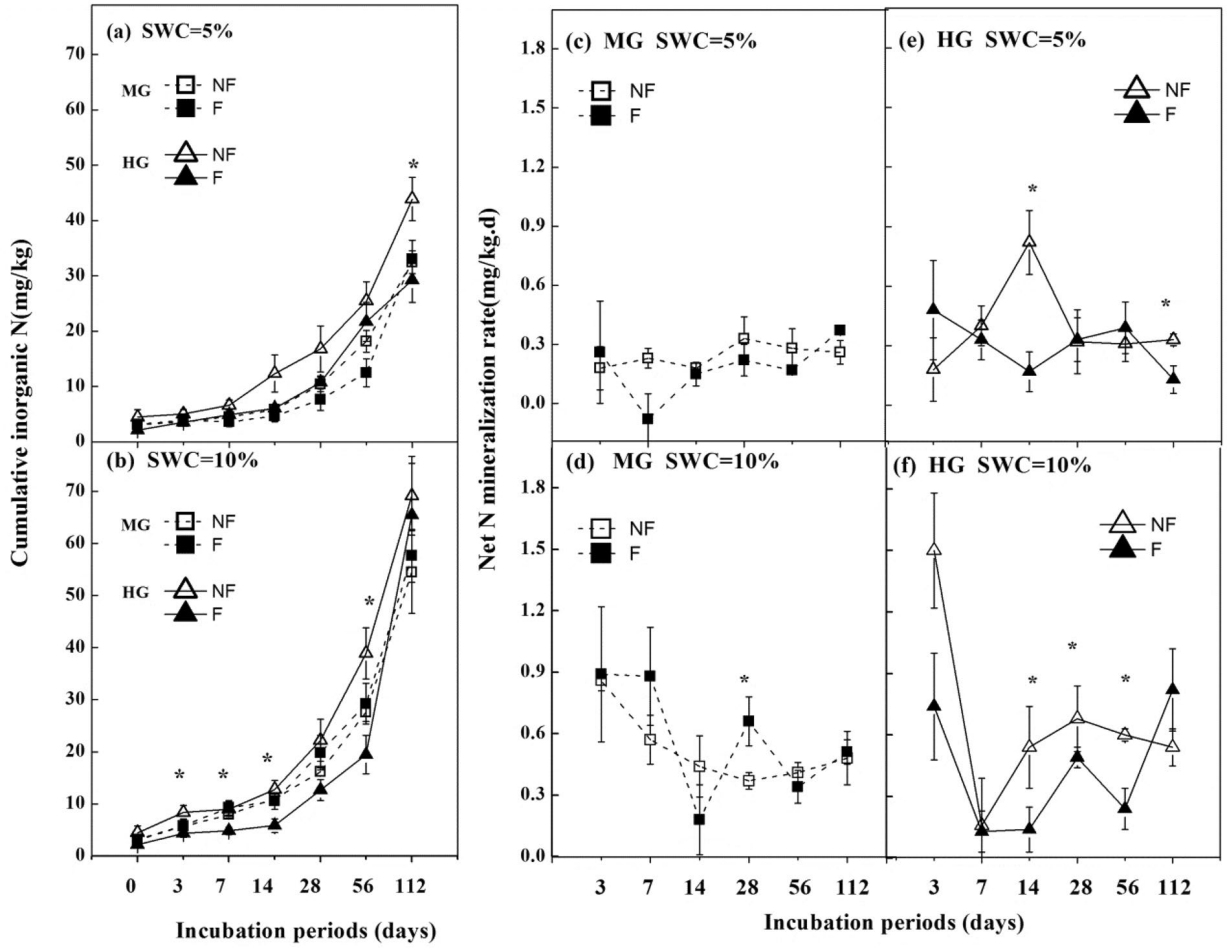
Soil cumulative inorganic N and net N mineralization rate (mean ± SE, n = 4) as affected by sites with different grazing intensity (MG and HG) and enclosure under different soil water contents (5% and 10%). *MG* moderately grazed site, *HG* heavily grazed site, *NF* non-fenced, *F* fenced treatment. ANOVA results are available in Table S2. Enclosure effects at a given incubation time are indicated above the data as: ***p < 0.001, **p < 0.01, *p < 0.05.

Compared with non-fenced treatment, the fenced treatment did not significantly change soil Nmin and Rate_Nmin at the MG site (Fig. 1a–d). Under the 5% soil water content level, enclosure significantly decreased the soil Nmin and Rate_Nmin on days 14 and 112 for the HG site, respectively (Fig. 1a,e); under the 10% soil water content level, the enclosure decreased the soil Nmin significantly and obviously decreased Rate_Nmin on days 14–56 for the HG site (Fig. 1b,f). The soil Nmin at the HG site was significantly higher than that at the MG site in non-fenced treatment under a 5% soil moisture level; however, the soil Nmin was not obviously different between the MG and HG sites in fenced treatments (Fig. 1a). The Nmin at the HG site was significantly higher than that at the MG site after 28 d in the non-fenced treatment; meanwhile, in the fenced treatment, the Nmin was significantly lower at the HG site than at the MG site on days 7–56 under a 10% soil moisture level (Fig. 1b).

### Quantification of ammonia-oxidizing microorganisms

**Figure 2:**
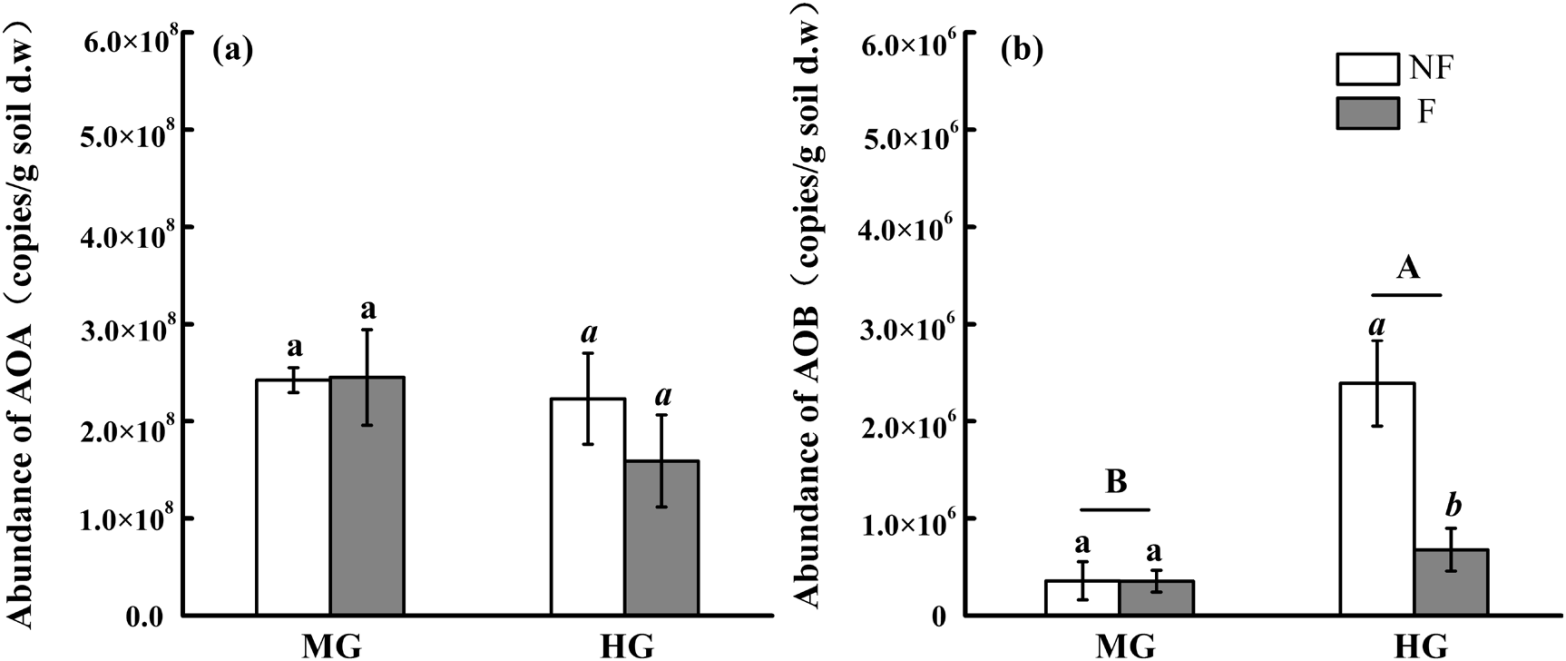
Effects of grazing intensity and enclosure on the abundance of AOA (**a**) and AOB (**b**) in soil. *MG* moderately grazed site, *HG* heavily grazed site, *NF* non-fenced, *F* fenced treatment. Different lowercase letters indicate differences between NF and F treatment at the MG and HG sites, respectively (*p* < 0.05, n = 4). Different capital letters indicate differences between the MG and HG sites across NF and F treatment (*p* < 0.05, n = 8).

High copy numbers of the *amoA* gene of AOA (approximately 2.5 × 10^8^ copies kg^−1^ dry soil) were detected in both the MG and HG sites, and they were two orders of magnitude greater than that of AOB in the two sites (Fig. 2a,b). For AOA, the *amoA* gene of AOA was not obviously different between the MG and HG sites in both fenced and non-fenced treatments (Fig. 2a). For AOB, compare with no-fence treatment, *amoA* gene copy numbers of AOB decreased significantly in fence treatment at the HG site but not obviously changed at MG site. The *amoA* gene copy numbers of AOB were significantly higher at the HG site than that at the MG site in both fenced and non-fenced treatments (Fig. 2b).

### Correlation analysis between the AOA and AOB abundance and soil physicochemical properties and PNR

A correlation analysis between abundance of AOA and AOB and soil physicochemical properties and PNR was used to identify the environmental variables which significantly control the abundance of AOA and AOB (Table 2). AOA was significantly and negatively correlated with pH (R = −0.52, *p* < 0.05). AOB was significantly and positively correlated with pH (R = 0.67, *p* < 0.01), NO_3_^–^N (R = 0.77, *p* < 0.01), NH_4_^+^-N + NO_3_^–^N (R = 0.65, *p* < 0.01), and C/N ratio (R = 0.52, *p* < 0.05) but significantly negatively correlated with TN (R = −0.53, *p* < 0.05) and N/P ratio (R = −0.58, *p* < 0.05).

**Table 2:** Correlation analysis between soil physicochemical properties and abundance of ammonia-oxidizing microorganisms.

	pH	SOC	TN	TP	C/N	N/P	C_NH4+-N_	C_NO3-N_	Available N	Moisture
AOA	−0.52*	0.35	0.3	0.13	−0.25	0.24	−0.17	−0.31	−0.29	0.04
AOB	0.67**	−0.37	−0.53*	−0.03	0.52*	−0.58*	0.3	0.77**	0.65**	−0.43

*Significant correlations at 0.05 level.

**Significant correlations at 0.01 level. Dates mean the relation coefficient.

**Figure 3:**
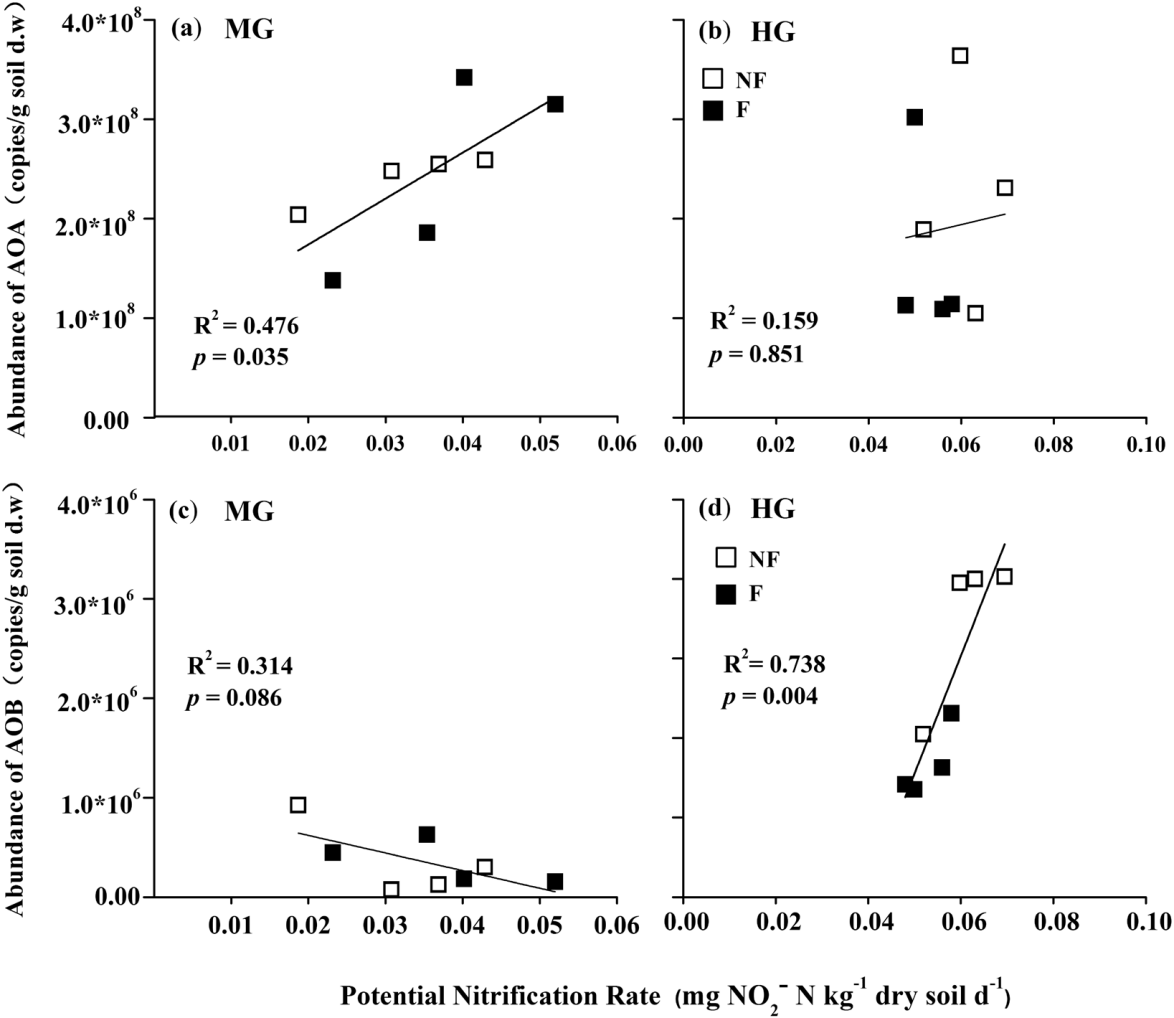
Relationships between potential nitrification rate (PNR) and the *amoA* gene copy numbers of AOA (**a,b**) and AOB (**c,d**) indifferent treatments. *MG* moderately grazed site, *HG* heavily grazed site, *NF* non-fenced, *F* fenced treatment. Single asterisk and double asterisk denote the significance at *p* < 0.05 and *p* < 0.01, respectively.

PNR was positively correlated with the AOA and AOB *amoA* gene copy numbers at the MG and HG sites, respectively (Fig. 3a,d).

### T-RFLP combined with cloning and sequencing for ammonia-oxidizing microorganisms

**Figure 4:**
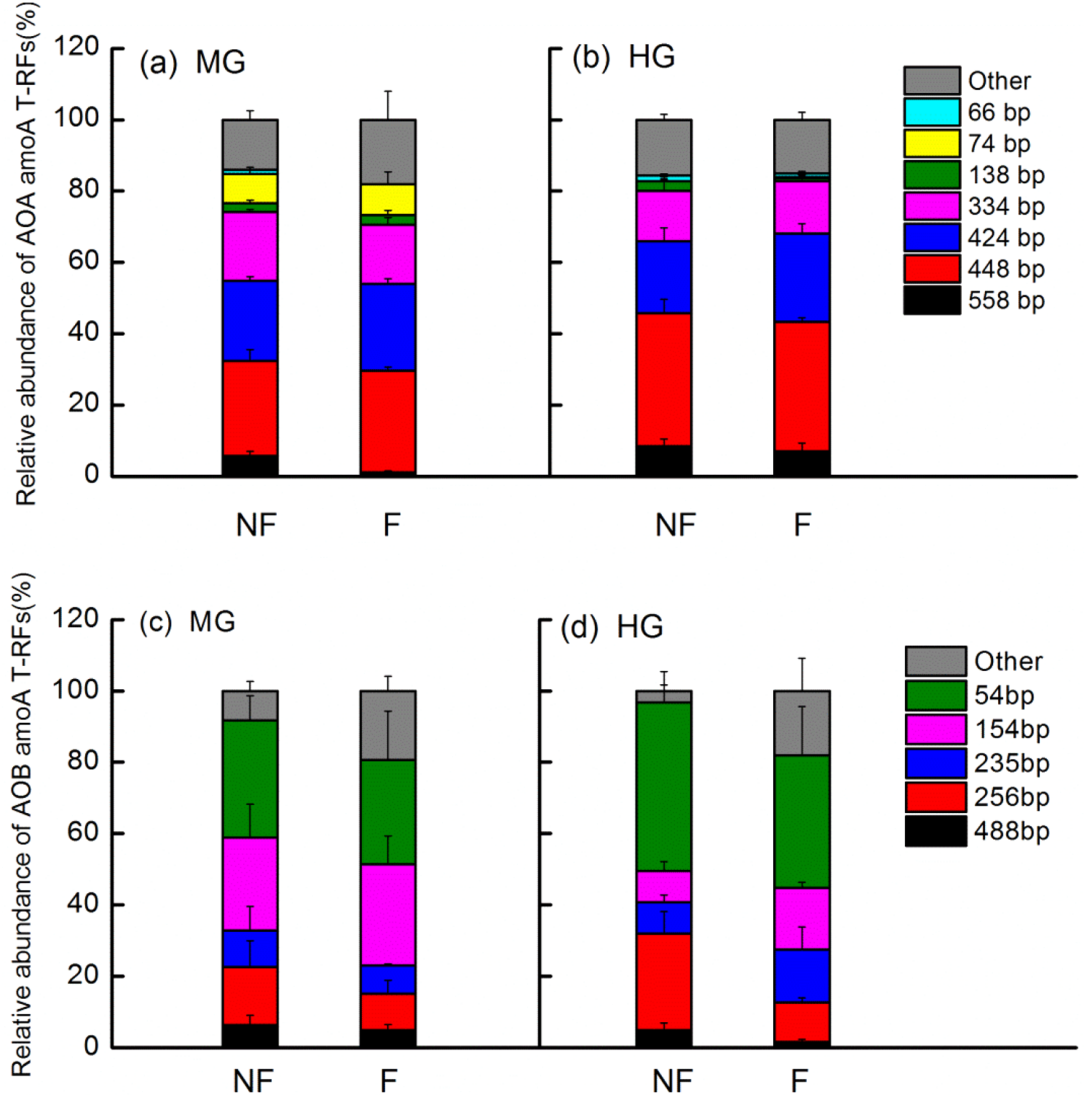
Terminal restriction fragment (T-RF) analyses targeting the functional gene *amoA* gene for AOA (**a,b**) and AOB (**c,d**) in soil. *MG* moderately grazed site, *HG* heavily grazed site, *NF* non-fenced, *F* fenced treatment. ANOVA results are available in Table S3 and Table S4. Correlation coefficient between soil physicochemical properties and T-RFs of AOB at HG site in Table S5.

T-RFLP analyses of AOA showed more T-RFs (seven to eight fragments) than that of AOB (six fragments) (Fig. 4). In both fenced and non-fenced treatments, the fragments of 558 bp and 448 bp of AOA significantly increased; however, the 74 bp fragment of AOA significantly decreased at the HG site compared with those at MG site (Fig. 4a,b; Table S3). The 154 bp fragments of AOB also significantly decreased at the HG site compared with those in both non-fenced and fenced treatments at the MG site (Fig. 4c,d; Table S4). At the MG site, the T-RFLP patterns of AOA and AOB showed almost no variation between fenced and non-fenced treatment (Fig. 4a,c). At the HG site, the T-RFLP patterns of AOA showed no significant variation between fenced and non-fenced treatment (Fig. 4b), however, the fragments of 256 bp of AOB decreased significantly in fenced treatment compared with those in non-fenced treatment (Fig. 4d).

**Figure 5:**
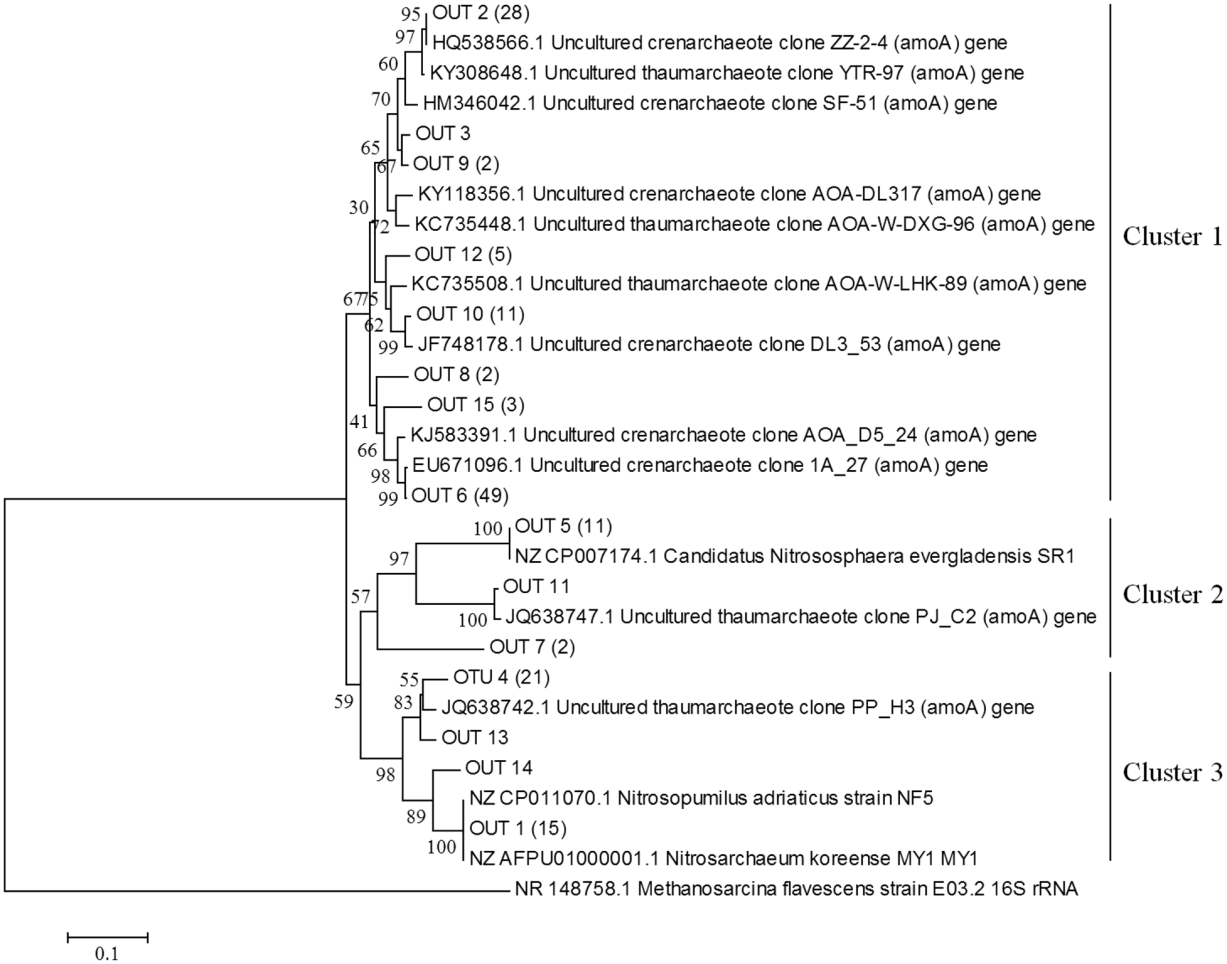
Neighbour-joining phylogenetic tree based on ammonia oxidizing archaeal *amoA* amino acid sequences. Clones with > 96% sequence similarity were considered as the same OTU and were named with OTU and numbers. Additional *amoA* sequences were obtained from the GenBank database. The scale bar represents 1% sequence divergence.

**Figure 6:**
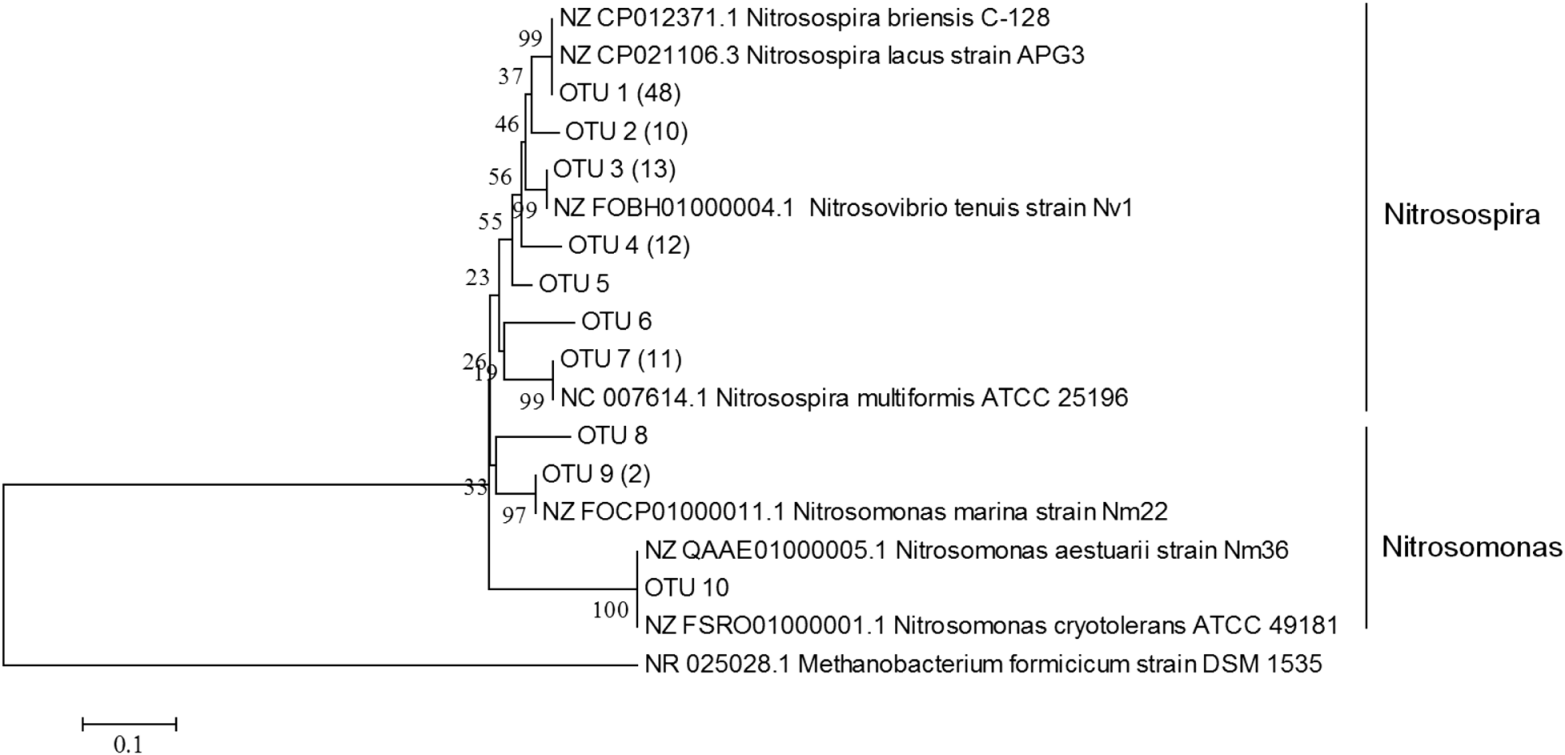
Neighbour-joining phylogenetic tree based on ammonia oxidizing bacterial *amoA* amino acid sequences. Clones with > 97% sequence similarity were considered as the same OUT and were named with OTU and numbers. Additional *amoA* sequences were obtained from the GenBank database. The scale bar represents 1% sequence divergence.

A total of 166 and 100 positive clones of *amoA* gene of AOA and AOB were randomly selected and sequenced, respectively (Figs. 5, 6). Based on deduced *amoA* amino acid sequences and reference sequences obtained from GenBank database, the neighbour-joining trees were constructed. Three clusters of the *amoA* gene of AOA, and AOB obtained from trees. Fifteen and ten OTUs were detected in AOA and AOB sequences, respectively. All AOA sequences in Cluster 1 had the highest similarity (95%-100%) with the *amoA* gene of AOA sequences obtained from uncultured crenarchaeote or thaumarchaeote (Fig. 5); Almost all the sequences in Cluster 2 belonged to *Nitrososphaeraceae*. There were fifteen and twenty-one sequences of the AOA *amoA* gene in Cluster 3, which belonged to *Nitrosopumilaceae*. The sequences of the *amoA* gene of AOB obtained in treated soil were affiliated with *Nitrosospira* and *Nitrosomonas* species, and most of the sequences were grouped into *Nitrosospira* species (Fig. 6).

## Discussion

### Effects of grazing intensity and long-term enclosure on Nmin

Our results indicated that the soil Nmin and Rate_Nmin at the HG site were higher than that at the MG site in typical grasslands of Inner Mongolia (Fig. 1, Table S2). It indicated that grazing promoted nitrogen mineralization and accelerated soil nitrogen turnover, such as alpine meadow in eastern Qinghai-Tibetan plateau in China, semi-arid grasslands on the Loess Plateau in China, and the Northern Great Plains of North America^12,21,26^.

Enclosures are an important measure for restoring degraded grassland because it affects soil fertility^19,27^. Our results indicated that after 10 years enclosure, the soil Nmin did not significantly change at the MG site; however, the Nmin and Rate_Nmin significantly decreased at the HG site (Fig. 1). According to Pan et al.^1,2^, ~ 70 to 90% of the N returned to the grassland soils via animal excreta. In addition, numerous studies found that the stability of soil aggregates would be improved after grazing stopped, thus increasing soil microbial immobilization of available nitrogen^10,28^. Therefore, reduces in animal excreta and microbial changes may explain the decrease of Nmin and Rate_Nmin at the HG site after enclosure. In contrast, studies in the Kobresia alpine grassland in Tibet and in the Hulunbeier grassland in Inner Mongolia showed that enclosure increased Nmin^14,29^, this may be because the enclosure induced changes in the composition of plant residue (e.g., higher cellulose, lower lignin/N ratio), which promoted decomposition and improved C release^27,30^, and a higher input of labile C promoted the reproduction of microorganisms after enclosure^31^. Therefore, the different soil physicochemical properties and microbial activity caused by enclosure measures impact soil Nmin accumulation.

### Effects of grazing intensity and long-term enclosure on the abundance of ammonia-oxidizing microorganisms and drivers for ammonia-oxidizing microorganism abundance

AOA abundance was two orders of magnitude greater than that of AOB in both MG and HG sites, which coincided with other grassland ecosystem^19,32,33^. AOB abundance at the HG site was significantly higher than that at the MG site (Fig. 2b). Enclosure significantly reduced AOB abundance compared with that under non-fence treatment at the HG site, however, enclosure did not obviously change AOB abundance at the MG site (Fig. 2b). The positive relationships were found between PNR and the abundance of AOB and AOA at the HG and MG sites, respectively (Fig. 3, Table 1), indicating that AOB and AOA may separately be the dominant functional players in ammonia oxidation processes at HG and MG site, because PNR was directly regulated by the ammonia oxidizers community^34^.

The differences in AOB and AOA abundances and/or community compositions between HG and MG sites may be due to their differential responses to soil environmental factors^35–37^. Soil pH were 7.17–7.41 and 7.97–8.15 at the MG and HG sites in this study, respectively. This indicated that HG increased soil pH by nearly 0.8 after treatment. Correlation analysis revealed that soil pH was positively and negatively correlated with the abundance of AOB and AOA, respectively (Table 2). AOA played an important role in nitrification in the temperate grassland of southwestern Germany with acidic soil (pH  5.7–6.9) and the typical steppe of Inner Mongolia with neutral soil (pH  7.13–7.61)^19,34,35^. AOB played an great role in nitrification in desert steppes of Inner Mongolia and plateau steppes in Wuchuan County with alkaline soil (pH  8.12–8.27)^14,36^. The abundance of AOB was positively and significantly correlated with soil NO_3_^–^N (R^2^ = 0.77, *p* < 0.05) and available nitrogen (NH_4_^+^-N + NO_3_^−^-N) (R^2^ = 0.65, *p* < 0.05), but there was no significant relationships between the AOA abundance and soil nitrogen in this study (Table 2). AOB adapted better to higher N environments, including high NH_4_^+^-N and NO_3_^–^N, whereas AOA were more favored by a low N environment^34,37–40^. There were also significant relationships between soil NO_3_^–^N and inorganic N concentrations and AOB abundance in grassland soil in southern England^41^. However, N availability alone is not the crucial factor controlling AOB abundance according to other studies^42^. The low C:N ratio was a primary factor regulating AOB abundance in Wessen et al. study^43^, which agrees with the study’s results, wherein, AOB abundance was significantly and positively correlated with soil C/N ratio.

### Effects of grazing intensity and long-term enclosure on the community structure of ammonia-oxidizing microorganisms

More T-RFs fragments (seven to eight) of AOA were observed compared with those (six T-RFs fragments) of AOB from T-RFLP (Fig. 4), indicating that the diversity of AOA was higher than that of AOB in our study. Grazing pattern was important in regulating the distribution of soil microorganisms and affected nitrification microbial communities by influencing soil physical and chemical properties (e. g. bulk density, NH_4_^+^-N)^1,2^. Enclosure of grassland from grazing affects microbial biomass and ammonia-oxidizing populations^17^. Compared with the MG site, the AOA and AOB community structures showed significant changes in the HG site. Enclosure conditions at the HG site significantly affected the community structure of AOB but not that of AOA compared with those under non-fence conditions (Fig. 4d). Enclosure measures increased soil physicochemical properties such as C/N ratio and NH_4_^+^ in the Haibei Alpine Meadow of Tibet, which significantly affected the AOA and AOB community structures^44^. The correlation analysis revealed that the T-RFs of 256 bp of AOB relative abundance was significantly positively correlated with NO_3_^–^N and NH_4_^+^-N + NO_3_^–^N at the HG site (Table S5). Therefore, the decrease of available nitrogen content entering the soil in the form of livestock excrement after enclosure at the HG site may explain the change in the AOB community structure (Table S5).

The phylogenetic analysis showed that three and two groups of the *amoA* gene of AOA and AOB were obtained from two trees. The sequences of AOA in Cluster 1 were affiliated with the *amoA* gene of uncultured crenarchaeote and thaumarchaeote. Nearly all the sequences in Cluster 2 belonged to *Nitrososphaeraceae*, and that in Cluster 3 belonged to *Nitrosopumilaceae* (Fig. 5). This coincided with early studies^19,45,46^, which found that most AOA sequences were affiliated with cluster *Nitrososphaera* (designated as I.1b AOA lineage^47^). Surprisingly, a *Nitrosopumilaceae* (designated as marine^47^) origin of AOA in our study was also found (Fig. 5).

The sequences of the *amoA* gene of AOB obtained in treated soil were affiliated with *Nitrosospira* and *Nitrosomonas* species, and most of the sequences were grouped into *Nitrosospira* species (Fig. 6). This indicates that *Nitrosospira* species are ubiquitous in the studied soil. *Nitrosospira* species as predominates in the AOB community were also found in studies targeting grassland soils^19,45,48^, arable soil^33^, and acidic upland soil^49,50^. However, Pan et al.^1,2^ and Olivera et al.^51^ detected a high number of *Nitrosococcus* lineages of AOB in different grazing intensity grassland soils, which is likely correlated with soil conditions caused by specific management measures. In addition, fifteen and ten OTUs were detected from AOA and AOB sequences, respectively, indicating that the diversity of AOA was higher than that of AOB in this study. Thus, *Nitrososphaeraceae* and *Nitrosospira* were the main AOA and AOB, respectively, regulating the nitrification process in this grassland soil.

## Conclusions

Enclosure significantly decreased the cumulative soil mineralized N and N mineralization rate at the HG site but not at MG site. Enclosure at the HG site significantly decreased the AOB abundance and changed the community structure of AOB. However, enclosure at the MG site did not significantly affect AOA and AOB abundance and community structures. PNR was positively correlated with the AOB and AOA abundance at the HG and MG sites, respectively. The abundance of AOA was significantly correlated with soil pH; however, AOB abundance was significantly correlated with several factors, such as soil pH, NO_3_^–^N, NH_4_^+^-N + NO_3_^–^N, C/N ratio, TN, and N/P ratio. The phylogenetic analysis showed that *Nitrososphaeraceae* and *Nitrosospira* were the main AOA and AOB, respectively, which may separately be the dominant influences on ammonia oxidation at the MG and HG sites and regulating nitrification processes in this grassland soil. In brief, enclosure measure in different grazing intensity grassland likely determined the niche special of ammonia-oxidizing microbes in the study soils through their effects on soil physicochemical properties and elements nutrient availability. The results of this study are of great significance for evaluating the ecological effects of enclosure in different grazing intensities.

## Supplementary Information

Supplementary Tables.

## Data Availability

All data generated or analyzed during this study were included in this published article.
