# Supplementary material for: Long-term enclosure at heavy grazing grassland affects soil nitrification via ammonia-oxidizing bacteria in Inner Mongolia
Source: Sci Rep. 2022 Dec 12;12:21464. doi: 10.1038/s41598-022-25367-z (PMC9744725; doi:10.1038/s41598-022-25367-z)
Supplement: Supplementary file 1 — Supplementary Tables. [file 41598_2022_25367_MOESM1_ESM.doc]

Table S1

Primer sets and thermal cycling condition used in the PCR analysis

| Target gene | Primer set | Sequence (5'-3') | Reaction system | Amplification procedure |
| --- | --- | --- | --- | --- |
| AOA-*amoA* | Arch-  amoAF Arch-  amoAR | STAATGGTCTGGCTTAGACG GCGGCCATCCATCTGTATGT | SYBR Premix Ex TaqTM 12.5μl, 1μl bovine serum albumin (25 mg ml^−1^), 0.5 μl each primer (10 μM), 2 μl diluted DNA | 95 ^o^C for30s; followed by 40 cycles of 94 ^o^C for 30 s, 53 ^o^C for 1 min, followed by 72 ^o^C for 30 s, and a final 1 min at 72 ^o^C |
| AOB-*amoA* | amoA-  1F amoA-  2R | GGGGTTTCTACTGGTGGT CCCCTCKGSAAAGCCTTCTTC | SYBR Premix Ex TaqTM 12.5μl, 1μl bovine serum albumin (25 mg ml−1), 0.5 μl each primer (10 μM), 2 μl diluted DNA | 95 ^o^C for30s; followed by 40 cycles of 94 ^o^C for 30 s, 58 ^o^C for 45 s, followed by 72 ^o^C for 45 s, and a final 45 s at 72 ^o^C |

Note: F is the upstream primer and R is the downstream primer

Table S2

Degrees of freedom and F-statistics from two-way ANOVA to assess the effects of grazing intensity, enclosure on the soil cumulative inorganic N and the average net N mineralization rates after specified incubation time

|  | Effect | *df* | 3 days | 7 days | 14 days | 28 days | 56 days | 112 days |
| --- | --- | --- | --- | --- | --- | --- | --- | --- |
| 5% | Cumulative inorganic N | |  |  |  |  |  |  |
|  | Grazed history (G) | 1 | 0.11 | 9.065* | 3.05 | 0.22 | 1.82 | 2.52 |
|  | Fenced (F ) | 1 | 0.29 | 3.57 | 3.38 | 0.17 | 0.02 | 0.7 |
|  | G*F | 1 | 0.1 | 1.46 | 2.73 | 0.31 | 0.98 | 9.249** |
|  | Net N mineralization rate | |  |  |  |  |  |  |
|  | Grazed history (G) | 1 | 0.12 | 7.26* | 12.87 | 0.23 | 1.9 | 1.61 |
|  | Fenced (F ) | 1 | 0.33 | 2.19 | 12.45 | 0.2 | 0.01 | 0.42 |
|  | G*F | 1 | 0.1 | 1.51 | 9.98 | 0.26 | 0.96 | 9.10* |
| 10% | Cumulative inorganic N | |  |  |  |  |  |  |
|  | Grazed history (G) | 1 | 0.12 | 9.786** | 0.04 | 0.39 | 0.4 | 2.06 |
|  | Fenced (F ) | 1 | 0.42 | 0.57 | 4.15 | 0.25 | 8.954* | 1.38 |
|  | G*F | 1 | 0.49 | 0.87 | 0.18 | 5.056* | 3.81 | 0.93 |
|  | Net N mineralization rate | |  |  |  |  |  |  |
|  | Grazed history (G) | 1 | 0.13 | 10.23** | 0.02 | 0.3 | 0.24 | 2 |
|  | Fenced (F ) | 1 | 0.46 | 0.35 | 4.74* | 0.21 | 7.66* | 1.31 |
|  | G*F | 1 | 0.49 | 0.86 | 0.17 | 5.18* | 3.9 | 0.95 |

Note: Significant level: * *p* < 0.05; ** *p* < 0.01; *** *p* < 0.001; no sign means *p* > 0.05.

Table S3

Degrees of freedom and F-statistics from two-way ANOVA to assess the effects of grazing intensity, enclosure on the AOA community structure

| Effect | *df* | 558 bp | 448 bp | 424 bp | 334 bp | 138 bp | 74 bp | 66 bp | Other |
| --- | --- | --- | --- | --- | --- | --- | --- | --- | --- |
| AOA community structure |  |  |  |  |  |  |  |  |  |
| Grazed history (G) | 1 | 5.33* | 11.78** | 0.18 | 2.7 | 0.43 | 26.35*** | 1.75 | 0.03 |
| Fenced (F ) | 1 | 2.42 | 0.004 | 1.71 | 0.13 | 0.63 | 0.11 | 2.88 | 0.17 |
| G*F | 1 | 0.77 | 0.25 | 0.25 | 0.54 | 0.96 | 0.01 | 0.35 | 0.3 |

Note: Significant level: * *p* < 0.05; ** *p* < 0.01; *** *p* < 0.001; no sign means *p* > 0.05.

Table S4

Degrees of freedom and F-statistics from two-way ANOVA to assess the effects of grazing intensity, enclosure on the AOB community structure

| Effect | *df* | 488 bp | 256 bp | 235 bp | 154 bp | 54 bp | Other |
| --- | --- | --- | --- | --- | --- | --- | --- |
| AOB community structure |  |  |  |  |  |  |  |
| Grazed history (G) | 1 | 1.5 | 1.4 | 0.3 | 6.50* | 1.34 | 0.48 |
| Fenced (F ) | 1 | 1.49 | 5.039* | 0.19 | 0.93 | 0.55 | 7.90* |
| G*F | 1 | 0.24 | 0.93 | 0.86 | 0.27 | 0.59 | 0.14 |

Note: Significant level: * *p* < 0.05; ** *p* < 0.01; *** *p* < 0.001; no sign means *p* > 0.05.

Table S5

Correlation coefficient between soil physical-chemical properties and T-RFs of AOB at HG site

| AOB | pH | SOC | TN | TP | C/N | N/P | C_NH_4_+-N_ | C_NO_3_--N_ | Available N | Soil  moisture |
| --- | --- | --- | --- | --- | --- | --- | --- | --- | --- | --- |
| 256 bp | 0.84** | -0.17 | -0.33 | 0.3 | 0.34 | -0.95** | 0.77* | 0.75* | 0.77* | -0.26 |
| 154 bp | -0.15 | 0.14 | 0.04 | -0.37 | 0.14 | 0.54 | 0.03 | -0.19 | -0.11 | 0.41 |
| 54 bp | 0.31 | 0.56 | 0.56 | 0.68 | -0.33 | -0.29 | 0.26 | 0.28 | 0.28 | 0.65 |

Note: * indicate significant correlations at 0.05 level; ** indicates significant correlations at 0.01 level. TN: Total N concentration, TP: Total phosphorus concentration, SOC: soil organic carbon concentration, C_NO3--N_: NO_3_^-^-N concentration, C_NH4+-N_: NH_4_^+^ -N concentration.
